# Factors Affecting the Applicability of Infrared Thermography as a Measure of Temperament in Cattle

**DOI:** 10.3390/vetsci12090913

**Published:** 2025-09-19

**Authors:** Paolo Mongillo, Elisa Giaretta, Enrico Fiore, Giorgia Fabbri, Bruno Stefanon, Lorenzo Degano, Daniele Vicario, Gianfranco Gabai

**Affiliations:** 1 Department of Comparative Biomedicine and Food Science, University of Padua, Viale dell’Università 16, 35020 Legnaro, PD, Italy; 2 Department of Animal Medicine Production and Health, University of Padua, Viale dell’Università 16, 35020 Legnaro, PD, Italy; 3 Department AgriFood, Environmental and Animal Science, University of Udine, 33100 Udine, UD, Italy; 4 Associazione Nazionale Allevatori Pezzata Rossa Italiana (ANAPRI), 33100 Udine, UD, Italy

**Keywords:** cattle, eye temperature, infrared thermography, stress, temperament, temperature–humidity index

## Abstract

Animal temperament, defined as consistent behavioral and physiological responses to stressors, plays a crucial role in cattle welfare, productivity, and safety during handling. This motivates researchers to identify objective, non-invasive methods for temperament assessment. Infrared thermography (IRT) has emerged as a promising tool to detect superficial temperature changes associated with stress and temperament in cattle. This study aimed to evaluate how superficial temperature variations measured by IRT in fattening bulls are influenced by environmental temperature, humidity, and temperament. The study involved 223 bulls at approximately 7.5 months old, while thermal images of eye and muzzle regions were captured at baseline and during restraint in a squeeze chute. Temperament was assessed using chute score and flight time, and environmental conditions were recorded via a temperature–humidity index (THI). Results showed significant increases in eye and muzzle temperatures during handling. Notably, changes in eye temperature were independent of environmental THI but correlated with flight time, with more temperamental bulls displaying larger temperature increases. In contrast, changes in muzzle temperature were strongly influenced by ambient THI and its variation at handling, consistent with the region’s thermoregulatory function. Temperament explained a small proportion of temperature variation. A follow-up experiment on a subset of 104 bulls around 11 months old showed no significant age effects on the IRT–temperament relationship. These findings indicate that IRT, particularly of the eye region, holds promise as a non-invasive, objective method to assess stress responses related to temperament in cattle. Careful selection of thermal regions and accounting for environmental influences are critical. While IRT alone accounts for limited variability, its integration with other behavioral and physiological measures could enhance temperament evaluation. This approach offers novel opportunities for improving animal welfare and management by identifying highly temperamental individuals without invasive procedures. Future research with higher temporal resolution and varied stressors is warranted to further elucidate temperature dynamics associated with temperament.

## Simple Summary

This study tested whether changes in surface temperature of cattle, measured with a thermal camera, can reveal how calmly or nervously they react to handling. Researchers worked with young bulls and recorded temperatures around the eyes and muzzle before and during restraint in a handling chute. At the same time, each animal’s behavior was measured by how calmly they stood in the chute and how quickly they left it afterward. The results showed that both eye and muzzle temperatures rose during handling. However, the two regions told different stories. Temperature changes around the eyes were linked to how excitable the bulls were: animals that rushed out of the chute more quickly also showed bigger increases in eye temperature. By contrast, temperature changes around the muzzle were mainly influenced by the surrounding weather conditions, suggesting that this area is more important for cooling the body. Although the increase in eye temperature only partly reflected differences in temperament, these findings suggest that thermal cameras could provide a useful, non-invasive way to monitor stress in cattle. The relationship held true for bulls between 7 and 11 months of age.

## 1. Introduction

Understanding the mechanisms by which animals respond to challenges and adapt to their environment is a current focus of farm animal science, following increased awareness that individual variability in behavior and physiology plays a crucial role in safety, productivity and welfare. One of the most relevant individual traits is fearfulness, which explains the animals’ tendency to react in a consistent way towards (perceived) threats, stressors and environmental challenges [1]. Operationally, fearfulness is measured as temperament, which is the quantifiable behavioral response of an animal to specific situations, such as human handling, restraint or exposure to novel objects [2]. Animals reacting more rapidly and/or vigorously to such situations are generally identified as ‘temperamental’ or having a ‘high’ or ‘strong’ temperament. The behavior of animals on the high end of the temperamental spectrum entails risks of injury for human operators, other animals and themselves [3]. Moreover, while temperament mainly refers to a behavioral reaction, underlying such reactions are neurophysiogical, endocrine and metabolic responses, which impact several aspects besides behavior [4,5,6]. Therefore, temperament is also associated with productivity, with more fearful and excitable animals yielding darker and tougher meat, having lower weight gain and lower dressing performance [7]. Finally, capturing the animals’ ability to cope with negative situations, and being, in essence, a reflection of a negative emotional experience, temperament is a relevant risk factor for both welfare and general health [8].

Several methods for the phenotypic quantification of temperament have been implemented. Some entail a subjective evaluation of the behavior of the animal under specific circumstances and according to standardized scales. For example, a ‘pen score’ is attributed after observing the behavior of an animal separated from its social group, taken to an individual pen and driven to a corner of the pen by a handler [9]. More practical alternatives take advantage of routine handling procedures, such as weighing. The ‘chute score’ entails the attribution of a score to the behavior of animals contained in a weighing chute, according to qualitative definitions, describing states of increasing physical agitation [10], typically from 1 (calm) to 5 (extremely agitated). A more objective and less demanding assessment of temperament in terms of human resources entails measuring the speed at which the animal covers a given distance along a raceway upon release from the weighing chute. The time taken to cover the distance—generally between 1.7 and 2 m—is then expressed as exit speed [11] or flight time [12]. The test relies on the assumption that less temperamental animals are slower in exiting the chute than more excitable and fearful ones. There is evidence that chute score and flight time are correlated, although to a different extent according to different studies, and that they are, at least in part, a reflection of the same trait. However, flight time may be a better methodology to evaluate cattle temperament, as its evaluation is objective, whereas subjective evaluations, such as the chute score, may allow for human error or bias to influence temperament evaluations [13]. The possibility to select for temperament requires the trait to be at least in part under genetic control. A recent meta-analysis reports that temperament, as measured by the chute score, is only moderately heritable [14]. Moderate heritability has also been found for exit velocity although with slightly higher estimates than chute score. Importantly, lower heritability has been found for exit velocity when tested at yearling than at weaning, suggesting that age at the time of testing might be an important factor to consider when evaluating this trait [14,15,16]. In fact, while both chute score and exit velocity have been found to be repeatable [17], variations in both are expected over time. Both maturity and experience are expected to shape the animals’ response to handling procedures, resulting in potentially lower scores when tested at an older age.

Temperament is not only a behavioral reaction but a reflection of a complex emotional response, with underlying physiological mechanisms. Two main neuroendocrine systems are associated with affective responses to perceived threat and more generally to stressors, i.e., increased activity of the sympathetic branch of the autonomic nervous system, which entails a generalized release of norepinephrine at nerve endings, affecting most organs and tissues throughout the body [18], and activation of the hypothalamic–pituitary–adrenal (HPA) axis, leading to the release of steroids from the adrenal cortex. Although both systems are implicated in animals’ response to stress, the typical proactive behavioral manifestations of temperament seem to be mainly associated with the rapid activity of the sympathetic nervous system. In line with this idea, several effects of adrenergic activity have been observed in temperamental animals, including changes in heart rate variability, modulation of the immune system and, of relevance to the present study, both general and local variations in temperature [18,19,20,21]. The release of catecholamines increases thermogenesis by mobilizing energy reserves and, potentially, by stimulating thermogenic response in lipidic tissue [18]. At the same time, the sympathetic nervous system determines a constriction of peripheral blood vessels, aimed at reducing heat loss and preserving energy and deflecting blood where it is needed more, e.g., skeletal and cardiac muscle and the central nervous system. Activation of the HPA axis also contributes to increasing body temperature, by increasing metabolic rate and enhancing the vasoconstrictive effects of the autonomic nervous system [22,23]. In accordance, within a few minutes of exposure to a stressor or threat, a generalized increase in core body temperature is observed, known as stress-induced hyperthermia (SIH) [24]. Thermal responses compatible with SIH have been observed in cattle undergoing handling procedures, supporting both the existence of the phenomenon in this species and the fact that handling is indeed perceived as a stressful event by cattle [19]. The same study reported an association between both the chute score and FS and maximal body temperature after handling, suggesting that individual variations in temperament are associated with different extents of changes in temperature.

However, measurement of core body temperature—typically performed through a rectal thermometer—is impractical during routine farm procedures, beyond representing per se a potential source of distress. To circumvent these limitations, attempts have been made to measure temperature remotely and in a non-invasive manner, characteristics that are allowed by infrared thermography (IRT). However, variations in superficial temperature do not necessarily reflect variations in core temperature [25]. In the first place, the vasoconstriction of peripheral arterioles induced by the sympathetic nervous system would imply a decrement, or, at the very least, a lower increment in skin than in core temperature. More complexity is given by the fact that superficial variations in temperature are time-dependent in both a body-region- and species-specific fashion. For instance, Stewart et al. [26] showed that temperatures in the eye region in cattle decrease immediately after exposure to a stressor, but increase above baseline a few minutes after. A similar time dependence was observed in the eye of blue tits [27], but in rats, a similar pattern was only observed in the tail, not the eye temperature [28].

It must also be noted that, being generally hairless and clean, the eye represents an optimal location for IRT and for these reasons, the interest in the possibility of applying eye IRT as a practical measure of an animal’s response to stressors has increased in recent years [29,30,31]. However, other regions might also be of interest in this regard. For instance, decrements in nose temperature have been observed in cattle after experiencing either a negatively or a positively valenced situation [32].

Finally, vasomotor responses are not merely a consequence of exposure to stressors. In fact, a primary driver of these physiological activities is the necessity for thermoregulation. Heat dispersion occurs mainly by increasing the flow of blood to the skin, which increases the delivery of metabolic heat that is produced by internal organs to the periphery from where it is lost to the environment by convection, radiation, and the evaporation of sweat from the skin or fluid from the upper respiratory tract [33]. Thus, changes in superficial temperature naturally reflect thermoregulatory needs, on top of stress-related responses, impacting the possibility to use superficial temperature as a direct measure of temperament. Furthermore, extreme environmental conditions of temperatures and humidity, particularly in their upper range, represent a well-known source of distress for cattle [34,35,36]; thus, thermoregulation and stress responses might interact with one another in determining vasomotor responses and consequent variations in superficial temperature. At present, however, the interaction between environmental conditions and temperament and its effect on changes in superficial temperature under stress are not yet fully understood.

The objective of the present study was to identify how superficial temperature variations in fattening bulls are influenced by both environmental factors and temperament. Although temperament reflects a consistent personality trait, behavioral and physiological responses are likely shaped by ontogeny and experience. Therefore, a secondary aim was to assess whether age affects the reliability of superficial temperature as an indicator of temperament by examining the stability of the relationship among superficial temperature, environmental temperature–humidity and temperament measures at two timepoints around sexual maturity. Ultimately, this information contributes to the overarching goal of determining whether changes in superficial temperature can reliably serve as proxies for animal temperament and how environmental factors must be accounted for when interpreting these temperature variations.

## 2. Materials and Methods

### 2.1. Experiment 1

#### 2.1.1. Farm and Management

This study was conducted at ‘Azienda Agricola Ricchieri’, Center for the Genetic Selection of Bulls of the Italian Simmental Breeder Association (ANAPRI), located in Fiume Veneto, northeast Italy (45.9250° N, 12.7323° E). The Italian Simmental is a dual-purpose breed, with around 200 male calves tested annually for growth performance to select breeding candidates. Calves from programmed breeding, sourced from ANAPRI associates, were brought to the Genetic Centre at approximately 30 days old. They were quarantined for a short period in a barn 1 km from the Centre, then weaned at 4 months. After weaning, calves were housed in pens of 6 individuals, in stall-slatted units. They had free access to water and received ad libitum a total mixed ration, distributed once a day between 7:30 and 9:00 h [37].

#### 2.1.2. Animals and Experimental Design

The study involved 223 fattening bull calves, with a mean ± SD age of 7.5 ± 0.5 months at the time of the study. Each bull underwent experimental procedures described hereafter once. Measures were taken from 8 to 18 bulls per study day, depending on the availability of animals of the appropriate age, for a total of 14 test days.

The study was conducted during routine bull weighing procedures to assess changes in superficial temperature in two anatomical regions, during the immobilization of the animal within a containment chute. The utilization of the chute also allowed the acquisition of standardized metrics of temperament: chute score and flight time.

**Figure 1:**
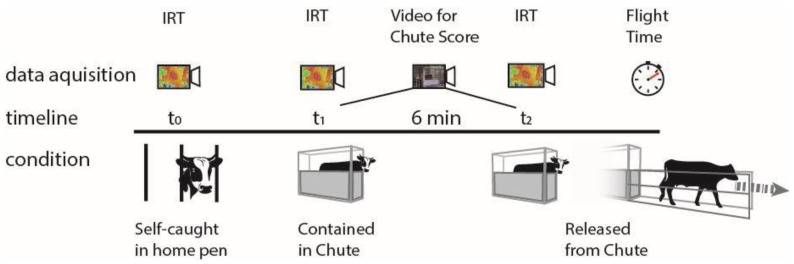
Schematic representation of the experimental activities. IRT = acquisition of infrared thermographic images.

Figure 1 provides a schematic representation of the experimental activities. For each subject, a set of thermal images was collected at a first timepoint (t_0_), while the bull was housed in its pen and contained in a self-catch head gate, just after being fed, between 9 and 10 AM. Next, bulls were individually taken out of their pen and contained in a squeeze chute in an adjacent hall, out of the sight of their pen mates, for weighing procedures. Upon entrance into the chute (t_1_), a second set of thermal images was taken. After 6 min (t_2_) from the beginning of containment, a last set of thermal images was taken, just prior to releasing the bull from the chute. Videos taken during the containment were later used to assess the bull’s behavior, obtain a chute score, and measure flight time, as described in detail below.

#### 2.1.3. Superficial Temperature

Thermal images were collected with an InfRec R550Pro thermal camera (Nippon Avionics Co. Ltd. Yokhoama, Japan) by standing or kneeling 1.5 m from the animal and level with the region of interest. Images were obtained from both eyes and the muzzle region. Several images per region were taken, making sure that the region of interest was on a plane perpendicular to the main axis of the camera.

Images were later reviewed with the software InfRec Analyzer, NS9500 Standard in order to eliminate those that were unusable (e.g., blurry, region of interest not visible, or not perpendicular to the main axis of the camera) and select two optimal images per region of interest. From images, temperatures were analyzed to obtain the maximal temperature of the muzzle region (MT) and the eye region (ET). These data were used to calculate the change in eye temperature upon entrance into the chute compared to baseline (⊗ET_1-0_) and after six minutes of containment (⊗ET_2-1_), and the same was performed for the muzzle region (⊗MT_1-0_; ⊗MT_2-1_).

#### 2.1.4. Chute Score

A camera (Xacti VPC-WH1EX, Sanyo, Osaka, Japan) was used to record the behavior of the bull during the 6 min of containment in the chute. Videos were used to compute a chute score, a qualitative score of the bull’s temperament, based on behavior expressed while contained, as described by [10]. Scores ranged from 1 (calm) to 5 (rearing and twisting of the body and struggling violently).

#### 2.1.5. Flight Time

A 2 m long corridor was created with metal railings at the exit end of the chute, so that upon release, the animal leaving was forced to walk in a straight direction, before being able to eventually walk back to its pen. A camera (Xacti VPC-WH1EX, Sanyo, Osaka, Japan) was used to record the behavior of the bull at the moment of its release. The video was used to obtain a measure of the time employed by the bull to walk across the 2 m corridor (flight time). To this end, the videos were imported into a software for the collection of behavioral data (The Observer XT 12.5, Noldus, Groeningen, the Netherlands), which was used to detect the moment when the bull trespassed the beginning and the end of the 2 m corridor.

#### 2.1.6. Temperature and Humidity Index

Environmental temperature and humidity were obtained from archives of the Regional Agency for Environmental Monitoring (ARPA) and referred to the nearest monitoring station, located approximately 1 km from the Genetic Centre (45.895661° N, 12.814989° E). Temperature and humidity were collected and used to calculate a temperature–humidity index for t_0_ (THI_0_) and t_1_ (THI_1_) for each bull, according to the formula THI  =  0.8 × T  +  RH/100 × (T − 14.4)  +  46.4. These data were used to calculate the difference in THI between t_1_ and t_0_ (⊗THI_1-0_). Since t_1_ and t_2_ were only spaced 6 min apart, it was assumed that the environmental temperature and humidity did not vary between these two sampling points.

#### 2.1.7. Statistical Analysis

A repeated-measures ANOVA was used to determine whether there were significant changes in maximal temperature in the eye or muzzle region across the three timepoints. The dependent variables for the analysis were ET in one model and MT in the second model, while the explanatory variable was the timepoint (t_1_, t_2_, t_3_). Bonferroni-corrected post hoc pairwise comparisons were run between timepoints, when a significant main effect was found for the timepoint factor.

General linear models were used to assess factors affecting changes in temperature in the eye and nose regions. The dependent variables used in the models were ⊗ET_1-0_, ⊗ET_2-1_, ⊗MT_1-0_, and ⊗MT_2-1_. Explanatory variables included in the initial models were THI_0_, ⊗THI_1-0_, flight time and chute score. First-order interactions between THI variables and both flight time and chute score were also included in the initial models. The models also included random terms, i.e., the season when sampling occurred, the number of the pen and the order by which animals were taken out of the pen, nested within pen number. A backward elimination approach was used for model selection, to maximize the variability explained by the models. Statistical analyses were performed with SPSS (Ver. 29, IBM, Armonk, NY, USA) and *p* < 0.05 was adopted for statistical significance.

### 2.2. Experiment 2

All sampling procedures were replicated, exactly as described for Experiment 1, on a subset of *n* = 104 bulls, approximately 14 weeks after the first sampling. At the time of the second sampling, bulls were on average 11.2 ± 0.9 months old.

Data were treated as presented in Experiment 1. These data were used to calculate correlations between measures taken in Experiment 1 and those taken about 3.5 months later, to assess reproducibility of the measures. Specifically, correlations were computed for FT, chute score, ET_0_, MT_0_, ⊗ET_1-0_, ⊗ET_2-1_, ⊗MT_1-0_, and ⊗MT_2-1_.

Moreover, a general linear mixed model was used to assess the effects of THI_0_, ⊗THI_1-0_, flight time and age on the increment in temperature between t_0_ and t_1_ and between t_1_ and t_2_, in both the eye and muzzle regions.

## 3. Results

### 3.1. Experiment 1

#### 3.1.1. Environmental Conditions

Throughout this study, environmental temperature ranged from −2.3 °C to 34.0 °C and humidity ranged from 36% to 100%, while the corresponding THI ranged from 36.3 to 84.3. Table 1 reports THI_0_ and THI_1_ values across different months of sampling and the number of animals sampled per month. It should be noted that for several months of sampling, samplings were performed on a single day, hence the lack of variability in THI_0_ (as thermographic samples were collected for all bulls at the same time on the given day). The same is true for THI_1_, although sampling at this timepoint entailed a longer procedure; therefore, about three hours between the first and last bull, and variability in THI between subjects was more probable.

#### 3.1.2. Flight Time and Chute Score

Walking through the 2 m exit corridor took a mean ± SD of 2.8 ± 2.4 s for a mean velocity of 0.97 ± 0.43 m/s.

Due to a damaged SD card, video recordings of the bulls’ behavior in the chute were unavailable for one day of sampling, resulting in 205 out of 223 bulls receiving an actual chute score. The distribution of chute scores is reported in Table 2. Because extreme scores were assigned to only a few individuals, bulls with scores of 1 and 2 were combined into a single group, as were those with scores of 4 and 5, for statistical analysis.

There was a significant but weak correlation between the flight time and the chute score (Spearman’s Rho = −0.16, *p* = 0.022).

#### 3.1.3. Superficial Temperature

**Figure 2:**
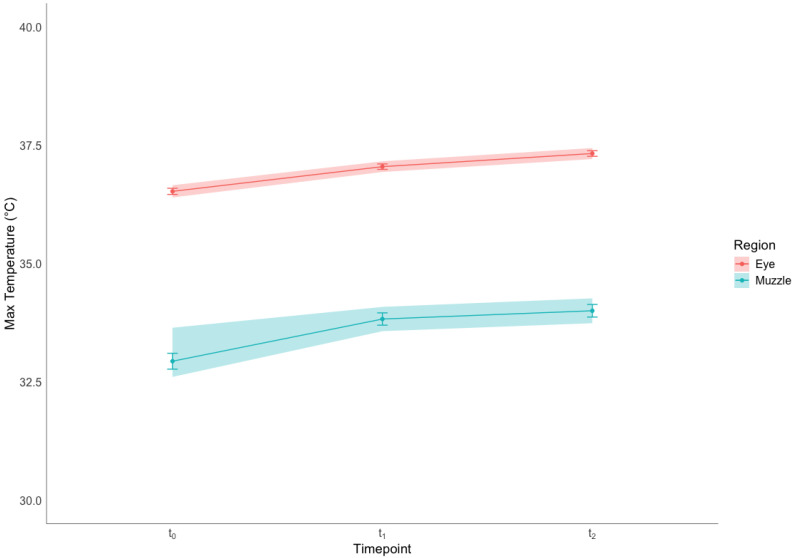
Mean ± SE and 95% confidence intervals (shaded areas) of the maximal temperature recorded in the eyes and the muzzle regions, before restraining in the squeeze chute (t_0_), immediately after being restrained (t_1_) and after 6 min of restraint (t_2_). Increments in temperature between t_0_ and t_1_ and between t_1_ and t_2_ were statistically significant for both the eye and nose regions (*p* < 0.001, Bonferroni-corrected post hoc pairwise comparisons after repeated-measures ANOVA).

Figure 2 shows the maximal temperature collected in the eye and muzzle regions at t_0_, t_1_ and t_2_. The temperature increased significantly across the three timepoints for both the eye region (F = 169.2, *p* < 0.001) and the nose region (F = 51.8, *p* < 0.001), and post hoc comparisons revealed that the increase was significant both from t_0_ to t_1_ and from t_1_ to t_2_ (*p* < 0.001 for all comparisons).

There were significant correlations between THI_0_ and the temperature collected at t_0_ in both the muzzle region (r = 0.69, *p* < 0.001) and eye region (r = 0.69, *p* < 0.001).

#### 3.1.4. Effect of Temperature, Humidity and Temperament on Increment in Eye and Muzzle Temperatures

The results of the GLMs assessing the effect of THI and temperament measures on the increment eye and muzzle temperatures are reported in Table 3. The change in muzzle temperature between t_0_ and t_1_ was explained by THI_0_, ⊗THI_1-0_ and flight time; specifically, the higher the THI_0_ and the smaller the difference with THI_1_, the smaller the increment in maximal temperature. As regards flight time, larger increments in temperature were found for faster bulls; opposite effects of flight time were found for increments in maximal temperature between t_1_ and t_2_, with larger temperature increments observed in slower bulls.

For the eye region, only flight time significantly explained the increment in temperature between t_0_ and t_1_, with smaller increments in temperature in slower bulls; none of the factors explained changes in temperature between t_1_ and t_2_.

The chute score had no significant effect on any of the variables.

### 3.2. Experiment 2

Correlations between measures collected during Experiment 1 and the same measures collected in Experiment 2 are reported in Table 4. High correlations (r > 0.5) were found for ET_0_, while moderate (0.3 < r < 0.5) correlations were found for MT_0_ and FT. Low but still significant correlations were found for ⊗ET_1-0_ and ⊗MT_2-1_, while no correlation was found for ⊗ET_2-1_ and ⊗MT_1-0_ and chute score.

The results of the models assessing the effect of THI, flight time and age on the change in temperatures in the eye and muzzle are reported in Table 5. For the muzzle region, increments in temperature between t_0_ and t_1_ were explained by ⊗THI_1-0,_ with larger increments in temperature observed with larger differences in THI. Changes in muzzle temperature between t_1_ and t_2_ were affected by THI, with larger increments observed for lower THI_0_, and FT, with larger temperature increments observed in slower bulls.

For the eye region, the increment in temperature between t_0_ and t_1_ was only explained by ⊗THI_1-0_, with larger increments observed with larger differences in THI. Increments in eye temperature between t_1_ and t_2_ were not affected by any of the factors.

Age and its interaction with THI_0_, ⊗THI_1-0_ and flight time had no effect on any of the parameters.

## 4. Discussion

### 4.1. Summary of Main Results

In the first experiment, significant increases in maximal superficial temperature of the eye and muzzle were observed in bulls removed from their pens and held in a squeeze chute, with further increases after six minutes of containment. Environmental temperature and humidity explained variations in temperature upon chute entry in the muzzle but not in the eye region. Flight time explained more variability: more temperamental bulls (i.e., those with shorter flight times) showed larger temperature increases in both regions immediately upon chute entry but smaller increases in muzzle temperature after six minutes compared to calmer bulls. Overall, temperament explained only a small portion of variability. A second assessment in a subset of bulls showed no age effects on temperature variations but consistent associations between temperament and temperature variations in the two regions.

### 4.2. Baseline Temperatures in the Eye and Muzzle Regions

Basal muzzle temperatures were approximately 3 °C lower and more variable than eye temperatures. While absolute values are hardly comparable across studies—as differences may arise from inherent characteristics of the instruments and their calibration—the extent of the difference between regions is consistent with other studies [38] and with the generally higher temperature and lower variability reported for the eye region than for other skin regions in cattle [39]. Baseline temperatures in both regions were positively correlated with environmental temperature and humidity. In agreement with these results, ref. [39] reported moderate to strong correlations between environmental temperature–humidity and temperature in the eye and other skin regions in cattle; similar effects have also been shown in humans [40]. Although the lack of a concomitant measure of rectal temperature does not allow us to determine how closely superficial temperature in either region reflects the animal’s core temperature, the larger variability observed in the muzzle likely reflects the region’s greater role in thermoregulatory responses, with circulation in the eye region more strictly constrained within regulated limits. Overall, the relationship between THI and IRT implies that absolute temperature values are hardly informative on anything but the physiological response of animals to environmental conditions, which could explain failures in previous attempts to correlate cattle’s temperament and eye temperature [21].

### 4.3. Effect of Restraint on IRT

Temperature variations, rather than absolute temperatures, are therefore more informative about the relationship between response to stressors and IRT. In this sense, a simple possibility to explain the increment in temperature observed in both the eye and muzzle regions when bulls were taken to and contained in the chute is movement-related thermogenesis. However, movement could only have contributed to a limited extent, if any, to the temperature increment: First, handling the bulls from the pen to the chute took about a minute in most cases and never more than 2 min, a time-frame which might be too short to determine a detectable increment in skin temperature [41,42,43]. Also, this would not explain why temperatures would still increase after 6 min of containment: while more agitated bulls showed substantial movement in the chute, if this impacted temperature, a significant effect of the chute score on temperature variations while contained would have been expected. All in all, this suggests that stress associated with handling, more than mere movement, impacted temperature variations.

The extent of the increment in eye temperature observed in the present study is comparable with that reported by others in cattle after stressors of different natures—for instance after transport [44], after being isolated from pen mates [38] or induced by handling [45], all of which produced increments in eye temperature in an approximate range of 0.5 to 1.0 °C. It is important to note that all of these studies measured temperature after several minutes of exposure to stressful events, while our data was collected at two timepoints—one immediately after being contained in a chute and one after 6 min—in the hope of having a better understanding of the time course of acute, stress-related changes in superficial temperature. The increment in temperature we recorded immediately after containment seems to contrast the notion that eye temperature decreases immediately after exposure to a stressor [26]. One potential explanation for this discrepancy is that, by the time bulls were moved from their pens into the chute, we had already passed the very short-term temperature drop phase and were instead observing the subsequent above-baseline, ascending phase. However, this explanation seems unlikely given the timeline of events: the process of moving bulls from their pens to the chute took less than two minutes, whereas previous studies report that stress-induced increases in eye temperature typically begin approximately five minutes after the initial exposure to a stressor. An alternative explanation should consider the entity of the stressor: the initial drop and the following increment in temperature reported by Stewart et al. [26] was only observed in bulls that were subjected to a painful procedure without anesthesia; conversely, bulls who received the same procedure with anesthesia only showed an increment 5 min post-treatment, without the initial decrement. A similar concept was clearly shown by Kuraoka et al. [46] who observed a drop in nasal temperature of macaques when exposed to either conspecific aggressive vocalizations or facial mimics, a higher decrement when the two cues were presented simultaneously, but no decrement when exposed to other non-threatening and likely less stressful communicative signals. Therefore, it is possible that the nature and the perceived intensity of the stressor impact the time course of the response and the detectable changes in terms of temperature. The bulls in our sample had already undergone handling and restraint a few other times before the present experiment and the procedure only involved being taken out of their pens and restrained in the chute, without other manipulations. Therefore, it is likely that the stress to which our bulls were subjected was only mild (see also [37]), justifying why we observed an increase rather than a decrease in eye temperature immediately after constraint.

### 4.4. Effect of THI and Temperament on Variations in Eye Temperature

The increment in eye temperature was not affected by either THI or by the difference in THI between sampling points, indicating that acute changes in eye temperature can be regarded as independent from environmental conditions. The significant effect of flight time indicates that the increase in eye temperature depends on the animal’s temperament. Specifically, shorter flight times were associated with larger increments in eye temperature, in line with expectations that behavioral manifestations of stronger emotional responses to stressors are accompanied by correspondingly greater physiological responses. Correlations between temperament and other physiological parameters in cattle exposed to moderate stressors have been reported before, including higher heart rate [1], higher insulin and insulin/glucose during insulin sensitivity test [47] and—of relevance—higher core temperature [19,48,49], in animals with faster exit times or higher chute scores. Despite the general consensus about the relationship between physiological variables and temperament, the effect of the latter on changes in eye temperature, and more generally in superficial temperature, is much less clear. For instance, Chen and colleagues did not find significant correlations between eye temperature and temperament [21], while Geburt et al. [50] reported a significant correlation between the two measures. Both studies, however, used a single sample point for eye temperature, which might be influenced by other variables, including THI. In this sense, this is, to the best of our knowledge, the first study reporting an association between temperament and variations in eye temperature in cattle. It remains to be clarified whether such a relationship reflects a generalized larger increment in temperature or a localized effect of a greater activation of the sympathetic nervous system in more temperamental animals. Moreover, it must be noted that the amount of variability explained by temperament is rather low, which calls into question whether increments in eye temperature could effectively be used as phenotypical proxies to characterize individual differences in response to stressors in cattle.

### 4.5. Effect of THI and Temperament on Variations in Muzzle Temperature

Increments in temperature were also found in the muzzle region. These were less expected, since decrements in muzzle temperature were previously reported for cattle exposed to either positive or negative emotional experiences [32]. However, the general parallelism with the trend shown by eye temperatures suggests that temperature is partly under the control of the same mechanisms in the two regions. One possibility to explain the increment rather than reduction in muzzle temperature is that the handling and restraint procedures were only moderately stressful for our subjects, as discussed above and as noted elsewhere [37]. Similarly to what was already discussed for the eyes, higher increments in temperature were observed in faster bulls, indicating that the extent of the changes in muzzle temperature reflects individual variations in cattle’s response to challenges. As opposed to the eye temperature, temperament also impacted the increment in muzzle temperature after 6 min of containment, but in the opposite direction: temperamental bulls showed smaller increments after 6 min of containment than calmer bulls. This seems to indicate that temperament impacts the speed, rather than the extent, at which temperature varies in this region. This aligns well with the idea that the perceived intensity of the stressor alters the time course of the response, whereby more intense distress leads to a faster onset, but potentially also a faster resolution of thermal responses.

As opposed to the eye region, increments in muzzle temperature upon restraint were also strongly impacted by THI, with greater increments when THI increased between baseline and restraint. The lack of an effect of THI on further increments in IRT during restraint indicates that the effects of environmental conditions on muzzle temperature had occurred before the animal was taken into the squeeze chute (baseline and restraint occurred up to 5 h apart). This aligns with the known role of the muzzle/nasal region in thermoregulation [51], with increased blood flow to the muzzle skin dissipating heat under warmer environmental conditions. Albeit to a smaller extent, THI_0_ alone also explained variations in muzzle temperature during containment, with smaller variations observed with higher basal THI. This might reflect either a lower effectiveness of vasomotor thermoregulatory mechanisms when environmental conditions are characterized by high THIs, or the lower stress-induced increment in temperature when basal superficial temperatures are already elevated. The different influences of THI on the eye and muzzle reinforce that while both regions exhibit temperature increases in response to handling stress, the underlying mechanisms driving the changes likely differ. For the muzzle, thermoregulatory demands seem to be superimposed on sympathetic activation, while changes in the eye temperature provide a more specific indicator of the systemic stress response.

### 4.6. Relationship Between Chute Score, Flight Time and Temperature Changes

Although both chute score and flight time are established indicators of temperament, the extent of their association varies widely across studies—from strong correlations to little or none [REF]—indicating that these measures reflect related but not identical behavioral and physiological traits. This notion is supported by our results, where the two measures were only weakly correlated, and where unlike flight time, the chute score did not explain any of the temperature variations. These results suggest that flight time is more directly connected to acute physiological responses triggered by confinement stress. Conversely, the chute score likely reflects a more complex and flexible behavioral response, which might be influenced by additional factors such as breed and prior handling experience. This interpretation aligns with the lack of correlation in chute scores observed between the two experiments of the present study. Furthermore, while chute score has been validated as a reliable temperament measure in various cattle populations, to our knowledge, it has not been specifically assessed in the Italian Simmental breed. Given the potential influence of breed on behavioral reactions to handling, a specific analysis of the validity of this measure in Italian Simmental bulls would be desirable.

### 4.7. Effect of THI and Temperament on IRT After Three Months

One of the aims of this study was to determine whether age/experience affects the viability of using superficial temperature changes as a measure of temperament in cattle. There was no effect of age at the moment of sampling (i.e., 7/8 or 10/11 months) on any of the variations in temperature between t_0_, t_1_ and t_2_, in any of the two regions. Of the significant factors found in the first experiment, only some were retained while others were lost. Overall, there was still an evident effect of environmental conditions, which explained not only variations in muzzle temperature, but in this case also in the eye region. Temperament measures still explained some of the variability in the muzzle temperature, but no longer explained that in the eyes. One possibility is that, considering the small effect that was already highlighted in the first experiment, the sample size for this second experiment was sufficient to highlight variations dependent on environmental conditions, but not those dependent on individual responses. A second possibility is that bulls might have become further habituated to the repeated handling procedures, thereby decreasing the role of the containment as a stressor while increasing the relative importance of environmental conditions on variation in superficial temperatures. Previous evidence indicates that habituation to repeated handling is evident from a behavioral standpoint [52] but not necessarily evident when looking at physiological parameters [53], and the fact that experience differently affects behavioral and physiological responses might explain the lack of effects of flight time on changes in IRT. However, it should also be noted that flight time was the variable for which higher correlations were found between the two age points—in agreement with previous evidence of moderate repeatability of such temperament measures [13]—while correlations were lower or non-significant for most of the IRT variables, suggesting that physiological responses (at least those responsible for changes in IRT) might also be affected by repeated handling. Finally, it is also possible that the relevance of thermoregulatory mechanisms increased with increased body mass, making IRT comparatively more dependent on environmental conditions as the animals grew.

### 4.8. Limitations

The present study has some limitations. Despite attempts to uniformly capture seasonal variations, certain environmental conditions—particularly those at the extreme high end of the temperature range—were underrepresented. Given the importance of elevated temperatures for bull welfare, a larger sample of data under such conditions would have been valuable. Additionally, it is possible that the lack of a relationship between chute score and temperature changes is partly due to the limited variance in chute scores; thus, greater variability in chute scores would have been desirable. However, bulls were not naïve to containment and prior experience in the chute may have dampened their behavioral responses. Furthermore, although exploratory analysis did not detect significant effects of the order by which bulls were taken to the chute on temperature or temperament measures, it cannot be completely ruled out that factors such as the time elapsed between baseline sampling and containment, or the time of day when containment occurred, influenced some of the responses.

## 5. Conclusions

This study demonstrates that infrared thermography is a promising non-invasive method for monitoring stress-induced superficial temperature changes in growing bulls, offering region-specific insights. Eye temperature reliably increased with temperament and mild handling stress in a way that was independent of environmental conditions, while muzzle temperature changes reflected both temperament and thermoregulatory responses, as indicated by their relationship with the temperature–humidity index. These findings suggest that proper selection of the thermal region and awareness of environmental influences are crucial to interpreting IRT data for temperament assessment.

Although temperamental measures accounted for a modest proportion of temperature variance, IRT can facilitate objective welfare monitoring and assist in identifying animals with heightened stress sensitivity, contributing to safer and more productive livestock management. Future research should focus on increasing measurement frequency, expanding age ranges, and integrating thermographic data with behavioral and physiological indicators to refine temperament evaluation and better inform welfare monitoring solutions.

## Figures and Tables

**Table 1:** Mean ± SD THI collected at t_0_ and t_1_ across the different months in which sampling was conducted and N of animals tested per month.

Month	N	THI_0_	THI_1_
1	25	51.2 ± 5.3	52.7 ± 6.2
2	29	57.3 ± 2.0	56.0 ± 5.3
3	18	57.4 ± 0.0	59.7 ± 2.6
5	17	74.5 ± 0.0	75.0 ± 0.0
6	18	77.6 ± 0.0	74.2 ± 4.8
7	18	80.1 ± 1.1	79.0 ± 1.8
8	18	82.9 ± 0.0	83.6 ± 0.0
9	17	67.9 ± 0.0	64.7 ± 2.1
10	18	68.1 ± 0.0	69.7 ± 0.0
11	18	61.1 ± 1.3	62.7 ± 1.6
12	27	50.0 ± 0.0	50.0 ± 2.2

**Table 2:** Distribution (N and % of the sample) of chute scores attributed to bulls of experiment 1.

Score	N	%
1	6	3
2	35	17
3	98	48
4	58	28
5	8	4

**Table 3:** Test statistics for general linear models assessing the effect of THI_0_, ⊗THI_1-0_, chute score and flight time on the increment of maximal temperature between t_0_ and t_1_ in the eye region and the nose region.

			Between-Subjects Effects		
Dependent Variable	Model R^2^	Factor	F	*p*	Partial Eta Squared
ΔMT_1-0_	0.652	THI_0_	7.78	0.006	0.064
		ΔTHI_1-0_	12.69	<0.001	0.101
		Chute Score	2.32	0.102	0.039
		Flight Time	9.65	0.002	0.079
ΔMT_2-1_	0.406	THI_0_	0.52	0.471	0.003
		ΔTHI_1-0_	2.28	0.132	0.017
		Chute Score	1.43	0.242	0.021
		Flight Time	5.22	0.023	0.038
ΔET_1-0_	0.490	THI_0_	1.23	0.269	0.009
		ΔTHI_1-0_	3.62	0.059	0.027
		Chute Score	1.06	0.347	0.016
		Flight Time	6.15	0.014	0.045
ΔET_2-1_	0.375	THI_0_	1.93	0.167	0.014
		ΔTHI_1-0_	0.31	0.576	0.002
		Chute Score	1.16	0.316	0.018
		Flight Time	0.01	0.969	0.000

**Table 4:** Pearson’s correlations between measures taken at the first sampling, around 7–8 months of age, and the same measures taken at the second sampling, at 10–11 months of age. Significant correlations are reported in bold.

	R	*p*
Flight Time	0.325	<0.001
Chute Score	0.083 ^1^	0.442
MT_0_	0.459	<0.001
ET_0_	0.785	<0.001
ΔMT_1-0_	−0.180	0.093
ΔMT_2-1_	0.218	0.027
ΔET_1-0_	0.245	0.015
ΔET_2-1_	−0.082	0.411

^1^ Spearman’s Rho.

**Table 5:** Test statistics for mixed linear models assessing the effect of THI_0_, ⊗THI_1-0_, flight time and age on the increment of maximal temperature between t_0_ and t_1_ in the eye region and the nose region.

Dependent Variable	Marginal Pseudo-R^2^	Factor	F	*p*
ΔMT_1-0_	0.038	THI_0_	0.66	0.417
		ΔTHI_1-0_	5.07	0.026
		Flight Time	0.63	0.426
		Age	3.18	0.076
ΔMT_2-1_	0.101	THI_0_	10.35	0.002
		ΔTHI_1-0_	2.05	0.153
		Flight Time	5.69	0.018
		Age	0.00	0.970
ΔET_1-0_	0.033	THI_0_	0.30	0.583
		ΔTHI_1-0_	4.71	0.031
		Flight Time	1.36	0.245
		Age	1.35	0.245
ΔET_2-1_	0.027	THI_0_	3.54	0.061
		ΔTHI_1-0_	1.90	0.170
		Flight Time	0.46	0.498
		Age	1.48	0.224

## Data Availability

The raw data supporting the conclusions of this article will be made available by the authors on request.
